# Tocotrienols Provide Radioprotection to Multiple Organ Systems through Complementary Mechanisms of Antioxidant and Signaling Effects

**DOI:** 10.3390/antiox12111987

**Published:** 2023-11-09

**Authors:** Stephen A. Shrum, Ujwani Nukala, Shivangi Shrimali, Edith Nathalie Pineda, Kimberly J. Krager, Shraddha Thakkar, Darin E. Jones, Rupak Pathak, Philip J. Breen, Nukhet Aykin-Burns, Cesar M. Compadre

**Affiliations:** 1 Department of Pharmaceutical Sciences, College of Pharmacy, University of Arkansas for Medical Sciences, Little Rock, AR 72205, USA; ujwani.nukala@fda.hhs.gov (U.N.); sshrimali@ualr.edu (S.S.); enpineda@uams.edu (E.N.P.); kjkrager@uams.edu (K.J.K.); shraddha.thakkar@fda.hhs.gov (S.T.); dejones@uams.edu (D.E.J.); rpathak@uams.edu (R.P.); breenphilipj@uams.edu (P.J.B.); naykinburns@uams.edu (N.A.-B.); 2 Tocol Pharmaceuticals, LLC, Little Rock, AR 77205, USA; 3 Joint Bioinformatics Graduate Program, University of Arkansas at Little Rock, Little Rock, AR 72204, USA

**Keywords:** tocotrienols, radioprotection, gamma-tocotrienol, delta-tocotrienol, radiation countermeasures, radiation injury, antioxidant, mechanism of action, signaling effects

## Abstract

Tocotrienols have powerful radioprotective properties in multiple organ systems and are promising candidates for development as clinically effective radiation countermeasures. To facilitate their development as clinical radiation countermeasures, it is crucial to understand the mechanisms behind their powerful multi-organ radioprotective properties. In this context, their antioxidant effects are recognized for directly preventing oxidative damage to cellular biomolecules from ionizing radiation. However, there is a growing body of evidence indicating that the radioprotective mechanism of action for tocotrienols extends beyond their antioxidant properties. This raises a new pharmacological paradigm that tocotrienols are uniquely efficacious radioprotectors due to a synergistic combination of antioxidant and other signaling effects. In this review, we have covered the wide range of multi-organ radioprotective effects observed for tocotrienols and the mechanisms underlying it. These radioprotective effects for tocotrienols can be characterized as (1) direct cytoprotective effects, characteristic of the classic antioxidant properties, and (2) other effects that modulate a wide array of critical signaling factors involved in radiation injury.

## 1. Introduction

There is a pressing need to develop radiation medical countermeasures to protect populations against radiological emergencies, including military personnel, civilians, and radiation workers [1,2,3,4,5]. In general terms, radiation countermeasure drugs are described as radioprotectors if they are administered before radiation exposure and as radiomitigators if they are to be used after radiation exposure [3,6,7,8]. The US Government is particularly interested in developing drugs that provide multi-organ protection against lethal radiation exposure [1,3,4,5,9]. Unfortunately, the current countermeasures available have considerable limitations and typically only protect one organ system, which places great urgency on developing suitable radiation countermeasures that provide whole-body protection [3,4,8].

In this regard, tocotrienols show promising potential as radioprotective countermeasures. Tocotrienols are currently under research and development to be used as a prophylactic agent for acute radiation exposure [9,10]. In animal models, tocotrienols are remarkably effective radioprotectors, providing powerful multi-organ protection to the hematopoietic system [11,12,13,14,15,16,17,18,19], gastrointestinal tract [12,13,20,21,22,23,24,25,26], and vascular endothelial tissue [12,13,15,27,28,29] against lethal doses of radiation [9,10]. Tocotrienols have the highest efficacy when subcutaneously pre-administered 24 h before radiation exposure. The greatest attention has been given to their ability to prevent acute radiation syndrome (ARS) in the hematopoietic system (H-ARS) [11,12,13,14,15,16,17,18,19] and gastrointestinal system (GI-ARS) [12,13,20,21,22,23,24,25,26], since treating these ARS subsyndromes is the top priority for radiation countermeasures.

**Figure 1:**
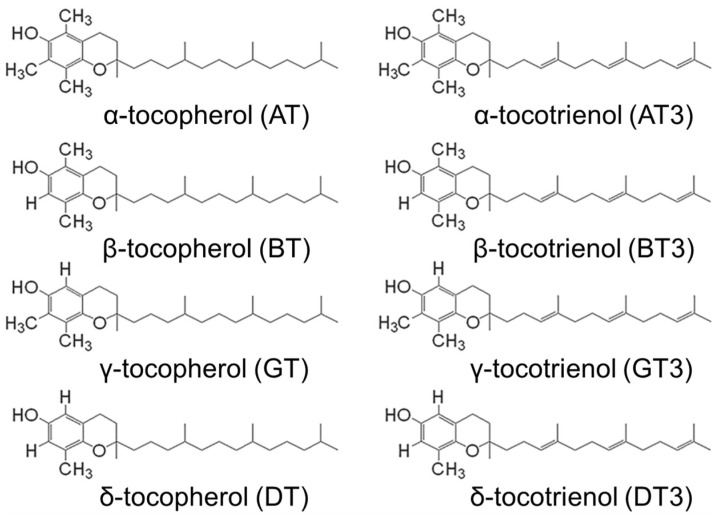
Chemical structures for the tocopherol and tocotrienol isoforms.

Tocotrienols are members of the vitamin E family and have potent antioxidant activity and other physiological effects, with a wide variety of potential therapeutic applications in numerous organ systems throughout the body [30]. The vitamin E family is comprised of tocopherols and tocotrienols. Tocotrienols possess an isoprenoid aliphatic side chain with an unsaturated farnesyl tail, whereas tocopherols have a saturated phytyl tail. There are four isoforms each for tocotrienols and tocopherols (α, β, γ, and δ), where they differ in the degree of methylation on the chromanol head (Figure 1).

Even though they are all classified as antioxidants, tocotrienols have substantially different PK/PD properties from tocopherols [30,31]. The four isoforms of tocotrienols function similarly to each other, but there are some important pharmacological distinctions [30]. Out of these isoforms, γ-tocotrienol (GT3) and δ-tocotrienol (DT3) have received the greatest level of attention for therapeutic applications, with GT3 being the most promising candidate to develop as a radioprotector [10,32].

In addition to their antioxidant properties, tocotrienols possess several physiological functions that tocopherols do not have, such as anti-inflammatory, anti-cancer, immune regulation, cholesterol-lowering, metabolic effects, and radioprotective effects [30,33,34,35,36]. Thus, tocotrienols have a wide range of potential therapeutic applications for almost every organ system and tissue in the body, including the cardiovascular, gastrointestinal, hematopoietic, renal, liver, brain, skin, and bone systems [30,33,34,35,36]. The common link between these investigated therapeutic indications is that they involve oxidative damage and/or inflammation, which are also heavily involved in radiation injury.

To further develop tocotrienols as radiation countermeasures, it is important to understand the mechanistic basis for their radioprotective ability in multiple organ systems against the effects of acute radiation syndrome. We have previously reviewed the basic antioxidant radioprotective properties of tocotrienols, focusing on the challenges to be addressed in developing them into radioprotective countermeasures [9]. Originally, it was thought that the radioprotective properties of tocotrienols were primarily due to their antioxidant effects—tocotrienols embed in membranes and scavenge ionizing radiation-induced free radicals. However, research over the past decade has revealed that tocotrienols also possess numerous signaling effects that are crucial for radioprotective efficacy that may not be derived solely from their antioxidant properties [12,15,21,22,37,38,39]. This raises the possibility that tocotrienols’ multi-organ radioprotective effects are due to a synergy of antioxidant effects and other signaling mechanisms.

## 2. Pharmacological Action of Tocotrienols

### 2.1. Antioxidant Effects

#### 2.1.1. Free Radical Scavenging

Tocotrienols exert their antioxidant effect by being inserted into cell membranes, with the farnesyl tail embedded within the lipid bilayer and the chromanol head facing the phospholipid head. Within the cell membrane, the tocols scavenge free radicals generated by ionizing radiation exposure or any other oxidative insult, thereby reducing oxidative damage to the membranes, specifically lipid peroxidation, or to other biomolecules in the vicinity. In cellular systems, tocotrienols possess superior antioxidant effects to their analogous tocopherol forms, likely due to how they interact with the cellular membrane microenvironment [40,41,42]. Biophysical studies comparing alpha-tocopherol (AT) and alpha-tocotrienol (AT3) show that AT3 has (1) its chromanol head situated closer to the membrane surface, (2) a more uniform distribution in the membrane, (3) a higher disordering effect on cell membranes, and (4) higher mobility within the lipid bilayer [40,41,42]. Therefore, it appears that tocotrienols exert changes to membrane architecture, distribution, and dynamics, which confer a greater ability to scavenge lipid radicals and a more efficient capacity for antioxidant recycling compared to tocopherols [42]. Another comparative study for all tocol isoforms found that tocopherols and their corresponding tocotrienols have the same antioxidant effects on lipid peroxidation in solubilized lipid membranes, but they have different effects on membrane uptake, fluidity, transfer, and architecture [43]. This supports the idea that the superior antioxidant efficacy of tocotrienols is likely due to how they biophysically interact within the membrane microenvironment.

#### 2.1.2. Induction of Antioxidant Enzymes

In addition to the direct radical scavenging effects, other studies show that tocotrienols induce the expression of key antioxidant enzymes such as superoxide dismutase, glutathione peroxidase, and NAPDH:quinoneoxidoreductase. The induction of antioxidant systems enables tocotrienols to exert antioxidant effects beyond the localized lipid microenvironment and to the rest of the cellular compartments [33]. This may explain how tocotrienols protect the entire cellular environment against oxidative damage from radiation exposure, including lipid peroxidation, protein ROS adducts, and DNA damage, even though tocotrienols are concentrated within membranes.

### 2.2. Modulation of Cellular Signaling Pathways

Tocotrienols directly and indirectly modulate numerous signaling pathways [30]. Key pathways relevant to the topic of radiation protection include inflammation, hematopoiesis, cell cycle, DNA damage and repair, metabolism, and mitochondrial bioenergetics.

**Inflammation:** Tocotrienols exhibit strong anti-inflammatory effects in stressed/damaged tissues by the inhibition of cytokines (interleukins, IFN-γ, TNF-α), transcription factors (NF-kB), and enzymes (COX-2), and these anti-inflammatory effects are central to many of the therapeutic application for tocotrienols [30].

**Hematopoietic cytokines**: Tocotrienols trigger hematopoiesis by the transient induction of hematopoietic cytokines, such as G-CSF and interleukins, which enables hematopoietic recovery after H-ARS [44,45]. These effects also likely have implications for other stressors and diseases involving immune functions.

**Transcription factors**: Tocotrienols activate NRF2 as part of its broadly cytoprotective mechanism [46,47]. NRF2 is a transcription factor with a pleiotropic cytoprotective role in protecting cells against oxidative stress and inflammation. NRF2 regulates the expression of antioxidant proteins, inflammatory factors, mitochondrial physiology, metabolism, immune responses, autophagy, and proteostasis [48]. The activation of NRF2 from tocotrienols may mediate the induction of key antioxidant enzymes, such as SOD, GPx, and NDH-2 [46,47]. Tocotrienols also modulate the expression of the transcription factor NF-kB, with differential effects depending on the context [30,49]. NF-kB is a “first-responder” transcription factor that is activated by stress, and it pleiotropically regulates inflammation, cytokine signaling, immunity, apoptosis, antioxidant defense mechanisms, and cellular/tissue regeneration. NF-kB is also a key regulator of the cellular response to radiation injury [50]. This suggests that the modulation of NF-kB by tocotrienols may be relevant to their radioprotective properties, though this has not been directly studied yet.

**Cell cycle regulators**: Tocotrienols modulate p53, which is a master regulator of the genome, cell cycle, DNA repair, cell survival, and apoptosis [51,52]. Due to its wide-reaching role as a master regulator, p53 is implicated in most types of disease and injury. Relatedly, a genomics study found that p53 was one of the most important factors in mediating the cellular and physiological responses to radiation injury [6,53].

**DNA damage/repair**: Tocotrienols attenuate DNA damage and promote repair through their antioxidant mechanisms in addition to the induction of DNA repair enzymes and related pathways, including RAD50, NRF2, and p53 [28,29,47].

**Metabolic effects:** Tocotrienols have cardioprotective metabolic effects by attenuating hyperlipidemia and hypercholesterolemia through its direct inhibition of HMG-CoA reductase by targeting it for degradation [54,55]. The hypolipidemic effects of tocotrienols synergize with their anti-inflammatory and antioxidant effects to protect against cardiovascular disease [56]. Inhibition of HMG-CoA reductase also provides broad vasculoprotective effects independently of cholesterol synthesis [57,58].

### 2.3. Mitochondrial-Protective Effects

Mitochondrial bioenergetic health is central to the pathophysiology underlying most types of chronic disease and acute injury. During stressors that lead to mitochondrial dysfunction, tocotrienols beneficially modulate the electron transport chain to improve oxidative phosphorylation, mitochondrial membrane potential, and ATP production [59,60,61,62,63,64]. Tocotrienols may also promote mitochondrial biogenesis through their effects on relevant transcription factors such as NRF2. The antioxidant properties of tocotrienols also directly protect the mitochondrial biomolecules from oxidative damage. Collectively, these mitochondrial-protective effects enable tocotrienols to mitigate stress-induced mitochondrial dysfunction, which may contribute to their numerous therapeutic applications. The mitochondrial effects of tocotrienols have only been investigated in two radioprotection studies so far and certainly merit further investigation [61,62].

## 3. Tocotrienols as a Potential Radioprotective Countermeasure

Tocotrienols first gained attention as potential radioprotective countermeasures when Ghosh et al. demonstrated that GT3 has powerful radioprotective properties in mice by protecting against life-threatening pancytopenia [11]. This seminal study set the stage for the following research into tocotrienols as radioprotective countermeasures. Shortly after, Li et al. demonstrated that another isoform, DT3, has similar radioprotective properties for the hematopoietic system in mice against lethal radiation doses [65]. Further studies found that both GT3 and DT3 provide remarkable multi-organ radioprotection for the hematopoietic, gastrointestinal, vascular endothelium, and other systems against life-threatening H-ARS and GI-ARS [9,10,32,66,67]. Both GT3 and DT3 appear to be providing multi-organ radioprotection through similar mechanisms of action. However, researchers at the Armed Forces Radiobiology Research Institute have mainly focused on GT3 as a candidate for development as a radioprotector [10,32,67].

Nearly all research in this domain has studied tocotrienols in the context of radioprotection, and so far there is only one published report that has investigated radiomitigation potential. Satyamitra et al. showed partial radiomitigation efficacy for 30-day survival when DT3 was subcutaneously administered to mice at 2, 6, or 12 h after lethal radiation exposure, but it was ineffective at the 24 h time point [18]. The lower radiomitigation efficacy of tocotrienols could be due to pharmacokinetic limitations or that their mechanism of action is simply more suitable for radioprotection [9]. However, there are efforts to improve the pharmacokinetics of tocotrienols to make them more effective radiation countermeasures, including the use of high-bioavailability formulations or the design of semi-synthetic derivatives with improved pharmacokinetic profiles, such as tocoflexols [68,69] or deuterated-DT3 [70].

Every tocotrienol radioprotection study covered in this review used a subcutaneous injection 24 h prior to lethal radiation exposure, ranging from a dose of 100–300 mg/kg in mice or 37.5 mg/kg in nonhuman primates. Thus, for this review article, shorthand statements such as “GT3 treatment” will accordingly refer to the subcutaneous 24 h pre-administration method, unless otherwise explicitly stated. All these studies utilized a mouse or nonhuman-primate model of total body irradiation (TBI) or partial body irradiation (PBI).

### Investigating the Multi-Organ Radioprotective Mechanisms of Action

Most tocotrienol radioprotection research has focused on their ability to protect the hematopoietic system [11,12,13,14,15,16,17,18,19] and the gastrointestinal tract [12,13,20,21,22,23,24,25,26], since treating H-ARS and GI-ARS are the top priorities for developing new radiation countermeasures. Tocotrienols also protect other tissues, such as the vascular endothelium [12,13,15,27,28,29] or the lungs [71], which is indicative of the truly whole-body radioprotection tocotrienols offer. These multi-organ radioprotective effects of tocotrienols are one of their most appealing properties as a potential radiation countermeasure. So far, no other approved radiation countermeasure has shown the ability to protect the whole body against acute radiation syndrome from lethal doses of radiation.

Many of these studies have investigated the radioprotective mechanisms for tocotrienols in multiple key organ systems beyond simply observing therapeutic endpoints. These radioprotective mechanisms of action for tocotrienols include their well-known powerful antioxidant effects, as well as other effects, such as the induction of hematopoietic cytokines and modulation of numerous signaling pathways. The “FDA Animal Efficacy Rule” for the regulatory approval of medical countermeasures has guided this tocotrienol radioprotection research [4,10,72]. One of its regulatory requirements is that the protective mechanism of action must be elucidated. We know tocotrienols are strong radioprotectors, but what is the mechanism? Is it simply its antioxidant and free radical scavenging effects, or are other signaling mechanisms also critically involved in their radioprotective mechanism of action? Further research is needed to comprehensively answer these questions, but the existing body of literature can provide a wealth of information and clues, which are covered in this review.

**Figure 2:**
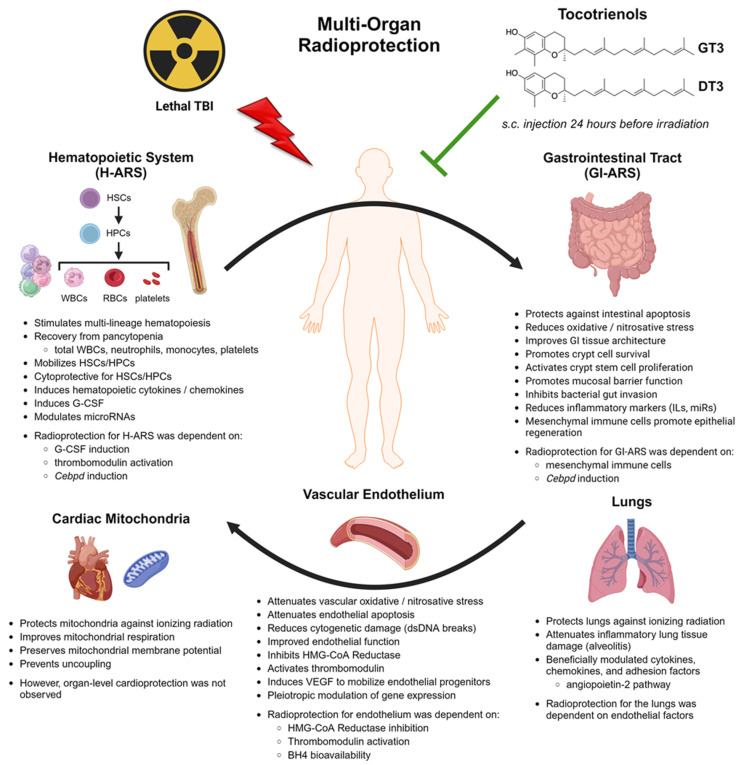
Schematic representation of the multi-organ radioprotective effects of tocotrienols, the underlying pharmacological mechanisms of action, and associated signaling pathways. The interdependency of each organ system is emphasized. Created with BioRender.com.

The radioprotective mechanisms of action for tocotrienols in each organ system are reviewed in detail in the following sections: the hematopoietic system (Section 4), the gastrointestinal system (Section 5), the vascular endothelium (Section 6), and other organ systems (Section 7). An overview of tocotrienols’ radioprotective signaling mechanisms is provided in Section 8, going beyond the established cytoprotective antioxidant effects. Figure 2 illustrates, from a systems biology perspective, all the observed therapeutic effects of tocotrienols and how the various mechanisms of action are implicated in a new paradigm of interdependent multi-organ radioprotection.

## 4. Radioprotection in the Hematopoietic System

The mechanism of lethality from H-ARS is through the destruction of the bone marrow and depletion of blood cells, pancytopenia, which causes fatal infection. Tocotrienols are effective at protecting against H-ARS, providing full survival and hematopoietic recovery against 100% lethal doses of total body irradiation [11,12,13,14,15,16,17,18,19]. With tocotrienol pretreatment, hematopoietic recovery of white blood cells is observed after radiation-induced cytopenia, particularly for neutrophils, which allows the body to fight off infections and survive H-ARS.

Tocotrienol-mediated hematopoietic recovery is a multi-stage process. Tocotrienols induce hematopoietic factors, which stimulate hematopoietic stem/progenitor cells (HSCs/HPCs) in the bone marrow to undergo proliferation and differentiation, yielding multi-lineage hematopoiesis and the recovery of white blood cell counts and platelets. Furthermore, tocotrienols mobilize HSCs/HPCs to the peripheral circulation due to their induction of hematopoietic cytokines and chemokines. Tocotrienols also promote survival and renewal of the bone marrow stem cells, which is crucial considering that ionizing radiation causes bone marrow cell death and myelosuppression.

This physiological cascade on the hematopoietic system involves a more complex pharmacological mechanism of action than the basic antioxidant properties of tocotrienols. These observed hematopoietic radioprotective effects from tocotrienols can be delineated into two major mechanistic categories: (1) Tocotrienol treatment induces hematopoietic factors, such as the cytokines G-CSF and various interleukins, as well as chemokines [13,14,15,21,37,38,39,44,45,73]. This effect appears to be a distinctly non-antioxidant mechanism of action. (2) Tocotrienols also directly exert cytoprotective effects on the HSCs and HPCs in the bone marrow, which enables their survival and renewal after cellular damage from ionizing radiation [13,14,19,37,65,74]. This cytoprotective effect of tocotrienols is most likely derived from its antioxidant effects, but other signaling mechanisms may indirectly contribute to bone marrow cytoprotection as well. Through this *synergistic* combination of antioxidant and other signaling effects, tocotrienols protect the bone marrow HSCs/HPCs and enable hematopoietic recovery after radiation-induced cytopenia. Tocotrienols also appear to exert radioprotective effects on multiple types of hematopoietic tissues, including bone marrow and the spleen.

### 4.1. Hematopoietic Factors

Cytokine panel screening shows that tocotrienol treatment induces an array of hematopoietic factors within 24 h, such as cytokines, notably G-CSF and various interleukins, along with chemokines [13,14,15,21,37,38,39,44,45]. These hematopoietic factors stimulate HPCs and HSCs to undergo proliferation and differentiation, yielding multi-lineage hematopoiesis. This effect is observed even in non-irradiated animals [39,44,45], which supports the hypothesis that the induction of hematopoietic factors is due to a different pharmacological signaling mechanism that does not derive solely from the cytoprotective/antioxidant properties of tocotrienols.

#### 4.1.1. Granulocyte Colony-Stimulating Factor

An early finding in tocotrienol radioprotection research is that tocotrienols induce hematopoietic cytokines, most notably granulocyte colony-stimulating factor (G-CSF) [13,14,15,21,37,38,39,44,45]. G-CSF is a cytokine/hormone glycoprotein that stimulates the bone marrow hematopoietic cells to proliferate and differentiate into granulocytes and then neutrophils [75]. G-CSF also mobilizes HSCs and HPCs into the peripheral blood [75]. The induction or administration of exogenous G-CSF treats radiation-induced neutropenia, which is key for protecting against H-ARS. To highlight the importance of G-CSF induction, the main radiomitigators in use are recombinant G-CSF and related analogues, such as filgrastim [3,6,8,76].

Co-administering G-CSF neutralizing antibodies with GT3 or DT3 negated the ability of tocotrienols to treat life-threatening neutropenia and enable hematopoietic recovery after H-ARS [37,38]. These experiments support the notion that the induction of G-CSF is critical for the radioprotective efficacy of both DT3 and GT3 [37,38]. It is unknown exactly how tocotrienols induce G-CSF and other hematopoietic cytokines, but it is likely due to a different mechanism than its antioxidant effects, considering (1) this effect is observed in non-irradiated animals [39,44,45] and (2) this effect is not generally observed in all antioxidants. Interestingly, a study found that very low doses of GT3 (25 mg/kg) still induced G-CSF [77]. The fact that doses of 200–300 mg/kg GT3 are required for radioprotection in animal models [10] suggests that G-CSF induction may be necessary but not sufficient for radioprotection.

#### 4.1.2. Other Hematopoietic Cytokines

In addition to the induction of G-CSF, tocotrienols exert differential effects on numerous other cytokines involved in hematopoiesis, inflammation, and immunity, such as interleukins. Tocotrienol treatment induces circulating levels of interleukins within 24 h, such as IL-1α, IL-1β, IL-6, and IL-9, which regulates hematopoiesis and enables recovery from H-ARS [15,37,38,44,45]. This effect is similarly observed even in non-irradiated animals [44,45]. At the tissue level, it appears that tocotrienols inhibit the levels of these same cytokines to protect against inflammatory tissue damage [25,26,71]. This highlights the differential impacts that tocotrienols have on cytokines—tocotrienols rapidly induce cytokines into the circulation within 24 h to trigger hematopoiesis [15,37,38,44,45], but they inhibit these same cytokines on the tissue level to protect against inflammatory damage from ionizing radiation [25,26,71]. Out of the numerous cytokines that tocotrienols modulate, most attention has been given to IL-1α, IL-1β, IL-6, and IL-9 in the context of radioprotection research.

#### 4.1.3. Hematopoietic Chemokines

Tocotrienols also induce several hematopoietic chemokines, such as MIP-1α, KC, and Mcp-1 [21,45]. Through chemokine receptor interactions, hematopoietic chemokines play a crucial role in the release and migration of mature immune cells into the peripheral circulation and the mobilization of HSCs and HPCs [78,79]. Chemokines are also involved in the survival and proliferation of progenitor cells, differentiation, and myelopoiesis [78]. Chemokines recruit inflammatory cells such as leukocytes to damaged or infected tissues as part of the innate immune response [80,81]. While the chemokine induction from tocotrienols has not been as thoroughly investigated as G-CSF and interleukins, it is likely they are involved in the radioprotective efficacy of tocotrienols for H-ARS.

### 4.2. Transcription Factor Cebpd

The transcription factor *Cebpd* is crucially involved in the radioprotective effects of GT3 on the hematopoietic system [21]. *Cebpd* is a transcription factor that regulates inflammation, oxidative stress, DNA damage, hematopoiesis, and immunity. Previous work has shown that knockout of *Cebpd* sensitizes mice to radiation injury and leads to lethal H-ARS from the depletion of HPCs/HSCs and subsequent pancytopenia [82]. In this study, the authors found that GT3 treatment in irradiated animals induces *Cebpd* and knockout of *Cebpd* attenuated the ability of GT3 treatment to recover levels of WBCs, particularly neutrophils, in addition to radioprotective effects on the GI tract [21]. While *Cebpd* is known to regulate hematopoietic cytokine expression, this study found that G-CSF was still robustly induced by GT3, even in *Cebpd* knockout animals. This suggests a G-CSF-independent mechanism for the role of *Cebpd* in mediating the radioprotective effects of GT3 on the hematopoietic system. The authors propose that GT3 is activating *Cebpd* through the NF-kB pathway or by modulating microRNAs [21]. These findings add *Cebpd* to the list of other transcription factors that tocotrienols therapeutically modulate [30].

### 4.3. MicroRNAs

Li et al. used a mouse model and human CD34+ hematopoietic cells to evaluate key radiation-induced inflammatory/stress and apoptotic signaling factors in multiple tissues and cell types, such as bone marrow, jejunum, liver, serum, and kidney [26]. They found that DT3 suppressed radiation-induced IL-1β and miR-30 signaling pathways in these tissues. Similar results were found in the human CD34+ hematopoietic cells, with knockdown experiments supporting the mechanistic conclusions that miR-30 is induced by the IL-1β to NF-κB pathway. The paper concluded that DT3 inhibits IL-1β-induced NF-κB signaling, leading to a downstream inhibition of radiation-induced miR-30. This DT3-mediated inhibition of miRNA-30 underlies the radioprotective efficacy of DT3 by attenuating apoptosis in these irradiated hematopoietic stem cells [26]. Two other studies found that GT3 has beneficial impacts on numerous microRNAs known to regulate the hematopoietic system’s response to radiation injury [52,83].

### 4.4. Direct Cytoprotective Effects

The cytoprotective effects of tocotrienol on HSCs and HPCs have also been characterized in several studies for both DT3 and GT3 in cell, mouse, and nonhuman-primate (NHP) models. In irradiated cell and animal models, tocotrienols promote cell survival and renewal for HSCs/HPCs in the bone marrow by preventing dsDNA breaks, preventing chromosomal aberrations, preventing apoptosis and cell death, promoting higher bone marrow cell count, preserving bone marrow cellularity, and preserving megakaryocytes [13,14,19,37,65,74]. These cytoprotective effects on the bone marrow cells are likely resulting from the antioxidant properties of tocotrienols, protecting them from ionizing radiation damage on the subcellular level. However, two studies using DT3 showed that the cytoprotective properties on bone marrow cells could involve other signaling mechanisms, such as the modulation of apoptosis pathways [19] and stimulating the Erk activation-associated mTOR survival pathway [65].

### 4.5. Summary of the Effects on the Hematopoietic System

**Figure 3:**
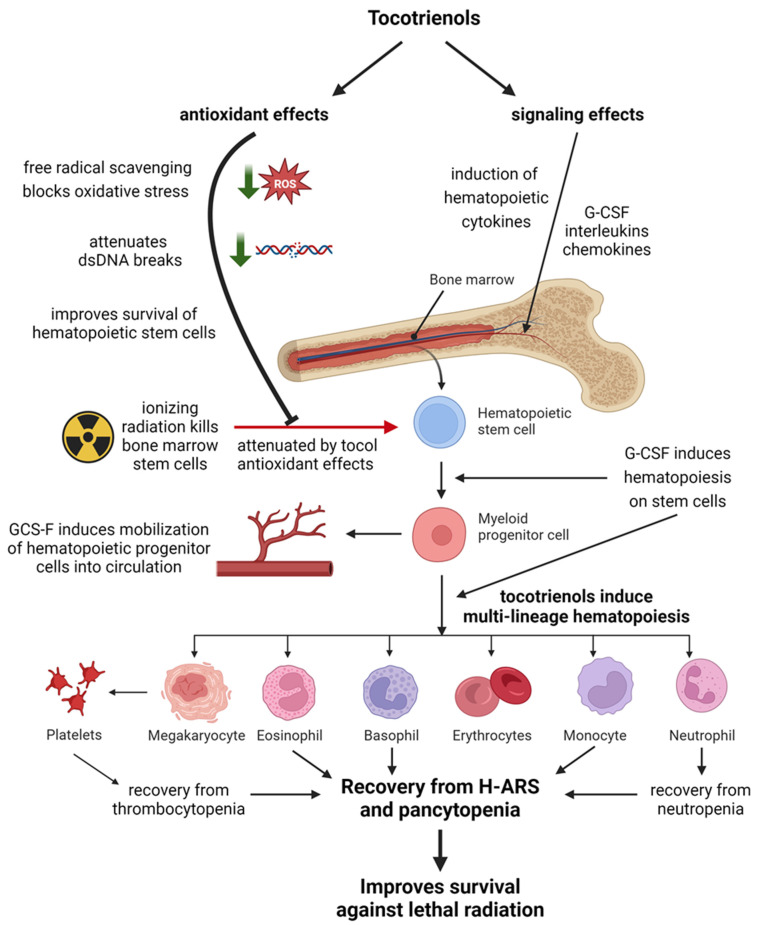
Schematic representation of how tocotrienols protect the hematopoietic system from radiation injury through complimentary actions of antioxidant and signaling effects. Created with BioRender.com.

A schematic demonstrating how tocotrienols protect the hematopoietic system against lethal radiation through complimentary mechanisms of antioxidant and non-antioxidant signaling effects is shown in Figure 3. A summary of the radioprotective effects of tocotrienols on the hematopoietic system is presented in Table 1.

## 5. Radioprotection in the Gastrointestinal Tract

GI-ARS causes death through the destruction of the gastrointestinal tissue. This is marked by intestinal epithelial cell death, loss of crypts, villi shortening, collapse of the gastrointestinal mucosal barrier, and general loss of gastrointestinal dysfunction. The loss of the mucosal barrier allows bacterial translocation, which causes lethal sepsis, especially since the concomitant H-ARS causes severe myelosuppression and pancytopenia.

Tocotrienols have been shown to protect against all of these parameters of GI-ARS, even up to supra-lethal doses of 10–15 Gy [12,13,20,21,22,23,24,25,26]. These radioprotective effects of tocotrienols include inhibiting apoptosis of intestinal tissue cells, promoting crypt cell survival, reducing oxidative and nitrosative stress, improving gastrointestinal tissue architecture, promoting mucosal barrier function, preventing bacterial translocation, preserving plasma citrulline levels, which is a GI function biomarker, accelerating mesenchymal immune cell recovery, epithelial regeneration, activating crypt stem cell proliferation, and reducing inflammatory markers [12,13,20,21,22,23,24,25,26]. Remarkable efficacy against GI-ARS has been observed in mouse models. However, only partial radioprotection efficacy has been observed in NHP so far, which highlights how much more challenging GI-ARS is to treat [10,23,24].

As in other organ systems, the radioprotective efficacy of tocotrienols in the GI tract is due to a combination of antioxidant and other signaling effects. Many of these radioprotective effects stem directly from the cytoprotective antioxidant effects, such as reducing intestinal cell death [12,13,21,22,23,24,25,26] and oxidative and nitrosative stress [21]. By attenuating oxidative damage and improving intestinal cell survival, tocotrienols help preserve the gastrointestinal tissue architecture, which maintains the mucosal barrier. The maintenance of the mucosal barrier is crucial for preventing lethal sepsis from bacterial translocation.

In the GI tract, the antioxidant and direct cytoprotective effects are the most apparent mechanisms of action for tocotrienols so far. In contrast, the non-antioxidant signaling effects of tocotrienols feature more prominently in protecting the hematopoietic system (Section 4) and vascular endothelium (Section 6) against radiation. Nevertheless, a few crucial signaling mechanisms have also been identified for the radioprotective effects of tocotrienols on the GI tract, most notably the mesenchymal immune cell mediated epithelial recovery mechanism [22], activating the transcription factor *Cebpd* [21], and reducing tissue inflammation [21,25,26].

### 5.1. Apoptosis Regulation

Tocotrienols have been shown to regulate apoptotic factors in the gastrointestinal tract of irradiated animals, which reduces apoptotic cell death [20,25]. One study found that GT3 treatment preferentially downregulates apoptotic factors and upregulates anti-apoptotic factors, which was supported by PCR and protein expression data. This resulted in a reduction of intestinal cell death in the jejunum [20]. Another study in irradiated mice found that DT3 inhibited intestinal cell apoptotic cell death by suppressing the pro-apoptosis stress-induced protein tyrosine kinase 6 (PTK6) [25]. In this case, it is tricky to disentangle whether the regulation of apoptosis from tocotrienols is a discrete signaling mechanism or whether it is simply downstream of the cytoprotective effect.

### 5.2. Mesenchymal Immune Cells

Garg et al. investigated whether the radioprotective effects of GT3 on the GI tract involve mesenchymal immune cells [22]. Specifically, the study was designed to see whether GT3 promoted gastrointestinal epithelial recovery through activating mucosal epithelial stem cells or sub-mucosal mesenchymal immune cells. Intestinal immune cells are crucial for protecting the gastrointestinal tract from injury, such as through irradiation. These intestinal immune cells also release signals that trigger intestinal epithelial cells to regenerate after injury. Hematopoietic injury from radiation leads to a severe depletion of these mesenchymal immune cells, which contributes to the pathogenesis of GI-ARS.

GT3 treatment attenuated intestinal cell death and improved intestinal structural integrity, as indicated by villus height and crypt depth. GT3 also attenuated radiation-induced intestinal permeability and restored mucosal barrier integrity, as indicated by the dextran uptake assay. GT3 also upregulated intestinal occludin levels, which is a crucial tight junction protein that regulates intestinal permeability. Next, the study found that GT3 promoted the recovery of neutrophils, macrophages, and lymphocytes in the intestinal tissue. Interestingly, GT3 treatment on irradiated animals did not promote the ability of crypt cells to form intestinal organoids ex vivo, which suggests that the mesenchymal immune cells are required for GT3 to promote regeneration of intestinal epithelial cells and tissue [22]. Mesenchymal immune cells are known to promote epithelial cell proliferation via AKT phosphorylation through the activation of the β-catenin pathway [85,86], and this study found that GT3 treatment enhanced this signaling pathway in the intestines from irradiated mice [22]. Notably, the ex vivo organoid experiment suggests GT3 does not directly act on epithelial stem cells to regenerate intestinal tissue after irradiation. The Garg et al. study concludes that GT3 protects the gastrointestinal tract from irradiation by improving cell survival, structural integrity, and barrier function through its radioprotective effects on mesenchymal immune cells [22]. Furthermore, GT3 activates the AKT phosphorylation signaling pathway in mesenchymal immune cells, which promotes epithelial regeneration [22]. The findings point towards a clearly multi-system signaling mechanism of action for protecting the gastrointestinal tract from radiation, which is also intertwined with the radioprotective effects of GT3 on the hematopoietic system and mesenchymal immune cells. This study adds the fresh perspective of a systems biology approach to understanding how tocotrienols provide multi-organ radioprotection.

### 5.3. Inflammatory Markers

Two studies using DT3 found that tocotrienols reduced several markers of inflammation in the gastrointestinal tract in irradiated mice [25,26]. DT3 treatment reduced the GI tract tissue expression of interleukins IL-1β and IL-6, which indicates anti-inflammatory effects. By attenuating these pro-inflammatory cytokines, less inflammatory immune cells would be recruited to the gastrointestinal tissue. By inhibiting IL-1β-induced NF-κB signaling, DT3 treatment leads to a downstream inhibition of radiation-induced miR-30 [26]. miR-30 is a microRNA that regulates stress responses, cell survival/death, and proliferation, and it has been implicated as a biomarker of radiation injury [26,87]. These findings highlight the differential impacts that tocotrienols have on inflammatory cytokines—tocotrienols rapidly induce interleukins into the circulation within 24 h to trigger hematopoiesis [15,37,38,44,45], but they inhibit these same cytokines on the tissue level, such as IL-1β and IL-6, to protect against inflammatory damage from ionizing radiation [25,26,71].

### 5.4. Transcription Factor Cebpd

Likewise, for the hematopoietic system, the transcription factor *Cebpd* was also found to be required for the radioprotective efficacy of GT3 on the gastrointestinal tract [21]. *Cebpd* is a transcription factor that regulates inflammation, oxidative stress, DNA damage, and immunity. GT3 treatment upregulates *Cebpd* in intestinal tissue, and even more so in the presence of radiation. Knockout of *Cebpd* in mice eliminated the ability of GT3 to protect intestinal crypt colony survival and attenuate oxidative/nitrosative stress. Surprisingly, GT3 *exacerbated* the gene expression of inflammatory cytokine and chemokine markers, such as IL-6, Mcp-1, and Cxcl1, and oxidative stress markers, including Nos2 and Hmox1, in the intestinal tissue of the *Cebpd* knockouts. These results show that without *Cebpd,* GT3 has a pro-inflammatory and pro-oxidative stress effect, rather than a radioprotective effect. The authors suggest that GT3 could be activating *Cebpd* through the NF-κB pathway or through microRNAs [21]. Overall, it appears GT3 is inducing *Cebpd* as a stress response factor to help multiple organ systems in the body recover from ionizing radiation.

### 5.5. Summary of the Effects on the Gastrointestinal Tract

A summary of the radioprotective effects of tocotrienols in the gastrointestinal tract is presented in Table 2.

## 6. Radioprotection in the Vascular Endothelium

The vascular endothelium is recognized as a major target of radiation injury [88,89]. Radiation damage to endothelial cells contributes to radiation pathology in many other key organ systems, such as bone marrow, gastrointestinal tract, liver, heart, kidney, skin, brain, and lungs [89,90,91]. These organs are all highly vascularized with endothelial cell-lined capillaries. Radiological injury to endothelial cells induces “endothelial cell activation, enhanced leukocyte-endothelial cell interactions, increased barrier permeability, and initiation of apoptotic pathways” [89]. These deleterious endothelium-mediated effects are particularly implicated in the pathophysiology of GI-ARS [89,90]. Extremely high radiation doses that degrade the vascular endothelium can even contribute to organ lesions [90,91]. Radiological damage to the endothelium also promotes the release of pro-inflammatory cytokines, which exacerbates organ-specific and systemic injuries [91].

The vast majority of studies have focused on the radioprotective effects of tocotrienols on H-ARS and GI-ARS; however, several studies have also demonstrated radioprotective effects on the vascular endothelium [12,13,15,27,28,29,39]. So far, only the GT3 isoform has been utilized in endothelium-related studies. These radioprotective effects on the endothelium systemically contribute to the multi-organ radioprotective effects of tocotrienols [15] since these organ systems are all vascularized and physiologically linked to the endothelium.

Similar to other organ systems, tocotrienols exert their radioprotective effects on the endothelium through a synergistic combination of antioxidant and non-antioxidant signaling effects. The radioprotective effects of GT3 on the endothelium include protecting against vascular oxidative and nitrosative stress [12,13,27], improving endothelial function [12], attenuating endothelial apoptosis [27], reducing cytogenetic damage by attenuating dsDNA breaks and inducing the DNA repair gene *Rad50* [29], inhibiting HMG-CoA reductase, activating the vasculoprotective factor thrombomodulin [12,13,15,92], and inducing endothelial progenitor cells [39,44]. A genomics study also found that GT3 modulated the gene expression pathways involved in oxidative stress, DNA damage, cell cycle, cell death, cell proliferation, and angiogenesis [28].

### 6.1. Oxidative and Nitrosative Stress

The antioxidant effects of tocotrienols are marked by decreased oxidative and nitrosative stress markers in endothelial cells after radiation exposure [12,13,27]. Tocotrienols may also help prevent eNOS uncoupling, which contributes to protection against oxidative and nitrosative stress [27]. The protection against cytogenetic damage of dsDNA breaks, as indicated by γ-H2AX foci, is also likely directly mediated by the antioxidant properties of GT3, in addition to the induction of the DNA repair gene *Rad50* [29].

### 6.2. HMG-CoA Reductase and Thrombomodulin

The most prominently studied non-antioxidant radioprotective mechanisms of tocotrienols in the vascular endothelium involve the inhibition of HMG-CoA reductase [12] and activation of thrombomodulin [15,92]. Both actions are well-characterized as a discrete pharmacological mechanism of action independent of free radical scavenging. Tocotrienols directly inhibit HMG-CoA reductase and target it for degradation through ubiquitinoylation, whereas statins are competitive inhibitors [54,55]. GT3 also induces thrombomodulin in both unirradiated and irradiated animals, likely through an HMG-CoA reductase inhibition-dependent mechanism [15]. Inhibition of HMG-CoA reductase with statins also leads to the activation of thrombomodulin through an eNOS-dependent mechanism [93,94], which highlights how these two vasculoprotective factors are functionally linked.

Blocking the effect of tocotrienols on HMG-CoA reductase and thrombomodulin were both shown to attenuate their radioprotective efficacy [12,13,15]. These HMG-CoA reductase and thrombomodulin pathways are also functionally linked in the context of radioprotection [92]. Inhibiting HMG-CoA reductase with statins is also associated with several vasculoprotective effects that are cholesterol-independent but eNOS-dependent, such as decreased oxidative stress, improvement of endothelial cell function, anti-inflammatory properties, and anti-fibrotic activity [57,58]. These same vasculoprotective effects were observed with GT3 treatment for irradiated animals [12]. One study found that combining simvastatin with GT3 enhanced radioprotective efficacy [92]. Thrombomodulin is an endothelial glycoprotein that has cytoprotective, anti-inflammatory, and anti-coagulant properties and regulates the cellular response to radiation injury [95,96]. Ionizing radiation impairs thrombomodulin levels, which exacerbates gastrointestinal tissue radiation damage [97]. GT3 treatment upregulates thrombomodulin, which provides vasculoprotective effects in irradiated animals [15,92]. In mice with mutant thrombomodulin, the protective effects of GT3 on survival and hematopoietic recovery were not observed [15]. These results highlight how the endothelium is connected to radiation injury in other key organ systems. This finding also shows how the radioprotective effect of tocotrienols on one system, the vascular endothelium, is critical for protecting another organ system, the hematopoietic system.

The attenuating effect of GT3 on radiation-induced vascular oxidative stress was reversed by co-administration of mevalonate, an HMG-CoA metabolic product [12]. This suggests that the antioxidant effects of GT3 in the vascular endothelium are not solely due to the free-radical scavenging effects of tocotrienols, but also involve an HMG-CoA reductase-dependent mechanism. This may be explained by the fact that HMG-CoA reductase inhibition induces eNOS to provide pleiotropic vasculoprotective effects [58]. Furthermore, the radioprotective effects of GT3 on the endothelium also appear to be mediated by increasing tetrahydrobiopterin (BH4) bioavailability, which attenuates oxidative/nitrosative stress and apoptosis in endothelial cells [27]. BH4 acts as a cofactor for eNOS and prevents its eNOS uncoupling, where it would produce superoxide instead of nitric oxide [27]. Collectively, these studies show that the antioxidant and signaling mechanisms of actions of tocotrienols are deeply intertwined for protecting the vascular endothelium against radiation injury.

### 6.3. VEGF and Endothelial Progenitors

GT3 treatment also induces vascular endothelial growth factor (VEGF) and endothelial progenitor mobilization in non-irradiated animals [39,44]. VEGF is a cytokine that acts as an endothelial survival factor and mitogen, induces mobilization of endothelial progenitor cells, and stimulates vasculogenesis and angiogenesis [98]. The ability of tocotrienols to induce VEGF and mobilization of endothelial progenitors may underlie their radioprotective and regenerative effects on the endothelium, and by extension, the other vascularized organs.

### 6.4. Summary of the Effects on the Vascular Endothelium

**Figure 4:**
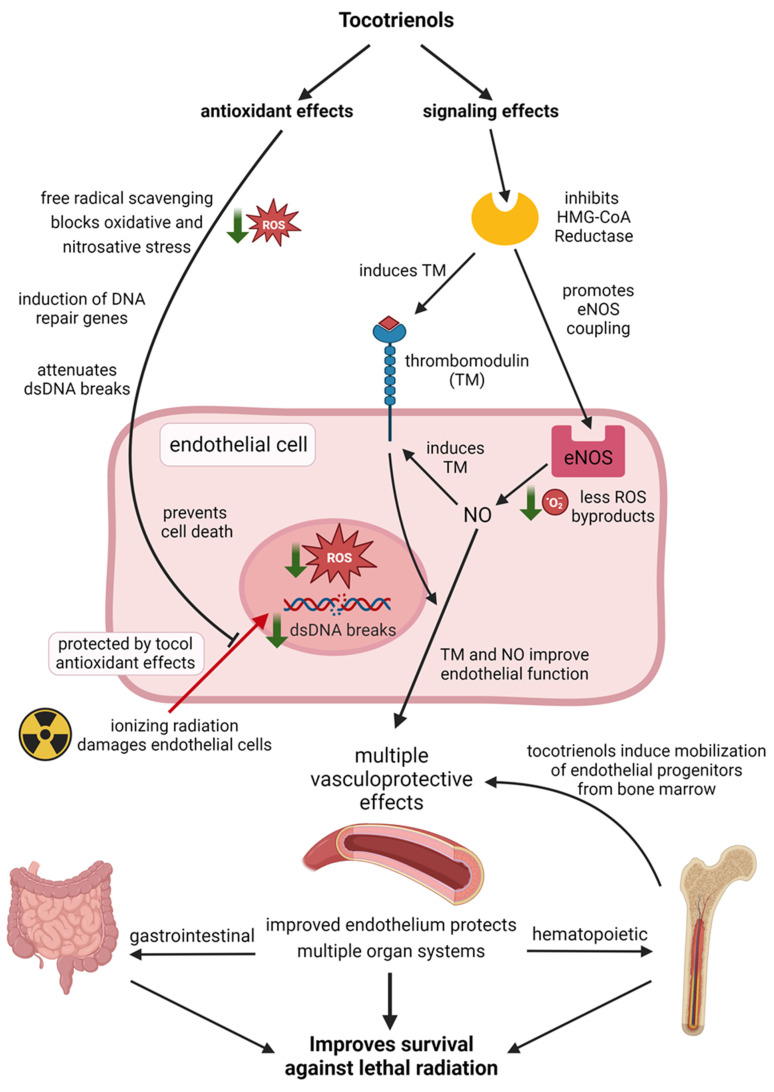
Schematic representation of how tocotrienols protect the vascular endothelium from radiation injury through complimentary actions of antioxidant and signaling effects. Created with BioRender.com.

A schematic demonstrating how tocotrienols protect the vascular endothelium against radiation injury through complimentary mechanisms of antioxidant and non-antioxidant signaling effects is shown in Figure 4. A summary of the radioprotective effects of tocotrienols in the vascular endothelium is presented in Table 3.

## 7. Radioprotection in Other Organ Systems

### 7.1. Lung

Kumar et al. performed an interesting study on the radioprotective effects of GT3 on the lungs [71]. This study utilized a targeted thoracic irradiation model to irradiate the lungs (lung-PBI) using extremely high doses of 14–16 Gy. Histopathological analysis showed that GT3 treatment protected the lung tissue from acute radiation injury, as indicated by the “combined alveolitis score”, with the greatest therapeutic effect observed at day 14 after lung-PBI. They evaluated a panel of dozens of inflammatory cytokines and chemokines that are implicated in pulmonary radiation injury. GT3 pre-treatment had differential and substantial impacts on the protein expression of three cell-adhesion molecules, VCAM-1, P-Selectin, and E-Selectin, along with six cytokines, Angiopoietin 2, myeloperoxidase 1, Flt3-L, CXCL9, CRP, and IGFBP5. Then, they conducted an extensive pathway analysis on Angiopoietin-2, which is an endothelial growth factor, and its downstream effectors. The pathway analysis showed that GT3 attenuated the detrimental effects of radiation on this Ang-2/Tie-2 to AKT-p/ERK pathway, which thereby promotes cell survival, angiogenesis, and vasculature in the lung. The radioprotective and anti-inflammatory effects of GT3 on the lungs appear to be mediated through complex signal transduction mechanisms involving endothelial factors, cell adhesion molecules, cytokines, and chemokines [71]. These findings also highlight the importance of the endothelium on the physiological health of the lungs, which are extremely vascularized organs.

### 7.2. Cardiac Mitochondria

Two studies have evaluated the radioprotective effects that tocotrienols have on cardiac mitochondria. One study utilized the H9c2 cell model and found that tocotrienol treatments, including a tocotrienol-rich extract from rice bran as well as pure DT3, prevented radiation-induced mitochondrial respiratory dysfunction and uncoupling [62]. The second study used a cardiac locally irradiated (21 Gy) mouse model and found that tocotrienols provided mitochondrial-protective effects, such as improving mitochondrial respiration, preserving mitochondrial membrane potential, and preventing opening of the mitochondrial permeability transition pore (MPTP) [61]. However, radioprotective effects were not observed on the organ level in regards to cardiac function and structure [61], despite their efficacy for cardiovascular disease and ischemic injury [56]. Regardless, these studies indicate that tocotrienols are also protecting mitochondria against radiation damage. The mitochondrial-protective effects of tocotrienols have not yet been studied in the context of the hematopoietic or gastrointestinal systems. However, this would certainly be worth studying further considering that mitochondria are severely damaged from ionizing radiation, which contributes to radiation injury pathophysiology [99,100].

### 7.3. Summary of the Effects on Other Organ Systems

A summary of the radioprotective effects of tocotrienols in other organ systems is presented in Table 4.

## 8. Overview of Radioprotective Signaling Mechanisms for Tocotrienols

The studies covered by this review have revealed that tocotrienols have numerous signaling mechanisms of action that are involved in their radioprotective effects, which may extend beyond their standard antioxidant properties. These include the induction of G-CSF, the induction of numerous hematopoietic cytokines and chemokines, mobilization of progenitor cells, anti-inflammatory action in injured tissues, HMG-CoA reductase inhibition, thrombomodulin activation, induction of DNA repair genes, induction of the transcription factor *Cebpd*, modulation of microRNAs, and beneficial effects on mitochondrial function. These effects are implicated in the radioprotective effects of tocotrienols in the hematopoietic, gastrointestinal, and vascular endothelial systems. The radioprotective signaling mechanisms of action observed for tocotrienols and their physiological implications are summarized in Table 5.

### Future Directions

The research reviewed in this paper clearly shows that tocotrienols provide multi-organ radioprotection through numerous signaling effects, in addition to their widely recognized antioxidant properties. However, this raises a major question—are these signaling effects entirely distinct pharmacological mechanisms from free-radical scavenging effects, or are they downstream effects of the antioxidant properties? A possible approach to differentiating the role of these potentially interrelated mechanisms is to utilize redox-silent analogues. For example, α-tocopherol succinate and 6-O-carboxypropyl-α-tocotrienol, which have no redox activity, have been shown to have anticancer signaling effects [101,102,103,104,105,106,107,108]. Some studies have also found that α-tocopherol succinate displays surprisingly strong radioprotective properties in mice, including in the hematopoietic system, which was dependent on G-CSF induction, and the gastrointestinal system [109,110,111,112]. However, no studies thus far have utilized redox-silent analogues of tocotrienols for radioprotection.

As shown in this review, the radioprotective effects of tocotrienols in ARS have been well established, along with extensive mechanistic research. However, their potential to protect against chronic radiation syndrome (CRS) or the delayed effects of acute radiation exposure (DEARE) have not been established and merits further investigation. Those studies could determine whether tocotrienols have broader clinical applications for radiation injury while revealing valuable information about their radioprotective mechanisms of action. Furthermore, there are many other organ systems involved in radiation injury that should be investigated in future tocotrienol radioprotection research, such as the heart, kidney, lung, and brain.

## 9. Conclusions

Although the antioxidant effects of tocotrienols are clearly involved in their radioprotection activity, their signaling effects are becoming increasingly implicated in their radioprotective efficacy in each organ system, including the hematopoietic, gastrointestinal, and vascular endothelial systems. The antioxidant properties and other signaling effects of tocotrienols appear to be mechanistically intertwined in the context of radioprotection, and some of these signaling effects may be distinctly non-antioxidant mechanisms. Furthermore, a systems biology perspective indicates that tocotrienols may be providing multi-organ radioprotection through interdependent physiological mechanisms between key organ systems. This proposed paradigm for the multi-organ radioprotective mechanisms of actions for tocotrienols, as depicted in Figure 2, suggests these compounds are particularly effective multi-organ radioprotectors due to a synergy of antioxidant effects and other signaling mechanisms.

## Figures and Tables

**Table 1:** Summary of observed radioprotective effects of tocotrienols on the hematopoietic system for acute radiation syndrome.

Hematopoietic System		
Major Radioprotective Effects of Tocotrienols for H-ARS	Model	References
Improves 30-day survival against H-ARS from lethal doses of total body irradiation Dose Reduction Factor for GT3: 1.29 Dose Reduction Factor for DT3: 1.27	Mice	GT3: [11] DT3: [18,65]
Stimulates multilineage hematopoiesis	Mice Primate	GT3: [11,16,17] DT3: [18,19]
Protects against pancytopenia Total white blood cells Neutrophils Monocytes Lymphocytes (DT3 only) Platelets	Mice Primate	GT3: [11,12,13,14,15,16,17] DT3: [18,19]
Particularly effective for neutropenia and thrombocytopenia	Mice Primate	GT3: [11,12,15,16,17] DT3: [18,19]
Induces hematopoietic cytokines/hormones/chemokines G-CSF Interleukins Chemokines	Mice	GT3: [13,14,15,37,44,45] DT3: [38,70]
G-CSF induction is required for radioprotective efficacy	Mice	GT3: [37] DT3: [38]
Promotes survival for HSCs and HPCs in the bone marrow Reduces cytogenetic damage Prevents dsDNA breaks Prevents chromosomal aberrations Prevents apoptosis and cell death Higher bone marrow cell count Preserves bone marrow cellularity Preserves megakaryocytes	Mice Primate hCD34+ cells	GT3: [13,14,29,37,74] DT3: [19,65]
Promotes spleen cell survival	Mice	GT3: [12,13]
Promotes recovery and proliferation of HSCs/HPCs	Mice	GT3: [37,74] DT3: [65]
Mobilizes progenitor cells into the peripheral circulation Hematopoietic progenitors Endothelial progenitors Stromal progenitors G-CSF- and VEGF-dependent	Mice without radiation	GT3: [39]
Reduces inflammatory and apoptotic markers IL-1β, IL-6, NF-kB, and miR-30 Inhibiting miR-30 attenuates radiation-induced apoptosis in hematopoietic cells	Mice hCD34+ cells	DT3: [26]
Modulates gene expression with microRNAs and modulates protein expression in mouse spleen	Mice	GT3: [52,84]

**Abbreviations**: gamma-tocotrienol (GT3); delta-tocotrienol (DT3); hematopoietic acute radiation syndrome (H-ARS); granulocyte colony-stimulating factor (G-CSF); interleukin (IL); hematopoietic stem cells (HSCs); hematopoietic progenitor cells (HPCs); double-strand DNA breaks (dsDNA break); vascular endothelial growth factor (VEGF); nuclear factor kappa-light-chain-enhancer of activated B cells (NF-κB); miR (microRNA); human CD34+ hematopoietic progenitor cells (hCD34+).

**Table 2:** Summary of observed radioprotective effects of tocotrienols in the gastrointestinal tract for acute radiation syndrome.

Gastrointestinal Tract		
Major Radioprotective Effects of Tocotrienols for GI-ARS	Model	References
Prevents apoptosis of intestinal tissue cells Upregulates pro-survival genes/proteins Downregulates apoptotic genes/proteins	Mice Primate	GT3: [20,23] DT3: [25]
Reduces GI-tract oxidative and nitrosative stress	Mice	GT3: [21]
Improves gastrointestinal tissue architecture	Mice Primate	GT3: [13,22,23] DT3: [25]
Promotes crypt cell survival	Mice	GT3: [12,13,20,21,22] DT3: [25]
Activates crypt stem cell proliferation	Primate	GT3: [23,24]
Promotes mucosal barrier function	Mice	GT3: [12,22] DT3: [25]
Inhibits bacterial gut invasion	Mice	GT3: [12] DT3: [25]
Preserves plasma citrulline levels	Mice	GT3: [12]
Accelerated mesenchymal immune cell recovery to protect the intestine Suppressed cell death, preserved villus height, increased crypt death, attenuated intestinal permeability, and upregulated occludin However, GT3 did not directly activate epithelial stem cells in organoids without immune cells Instead, GT3 activated mesenchymal immune cells to release signaling factors that regenerate the intestinal epithelium Mediated through a β-catenin to AKT phosphorylation pathway	Mice	GT3: [22]
Reduces GI-tract inflammatory markers IL-1β, IL-6, and miR-30	Mice	GT3: [21] DT3: [25,26]

**Abbreviations**: gamma-tocotrienol (GT3); delta-tocotrienol (DT3); gastrointestinal acute radiation syndrome (GI-ARS); gastrointestinal tract (GI-tract); interleukin (IL); miR (microRNA).

**Table 3:** Summary of observed radioprotective effects of tocotrienols on the vascular endothelium.

Vascular Endothelium		
Major Radioprotective Effects for Tocotrienols	Model	References
Protects against vascular oxidative and nitrosative stress Dependent on HMG-CoA reductase inhibition	Mice	GT3: [12,13,27]
Inhibits HMG-CoA reductase Provides vasculoprotective effects against radiation	Mice	GT3: [12]
Induces thrombomodulin Contributes to radioprotective efficacy for H-ARS and survival	Mice	GT3: [15,92]
Attenuates endothelial apoptosis	Mice	GT3: [27]
Reduces cytogenetic damage Prevents dsDNA breaks, as indicated by γ-H2AX foci Induces DNA repair genes, such as *Rad50*	HUVEC cells	GT3: [29]
Improved endothelial function Dependent on HMG-CoA reductase inhibition	Mice	GT3: [12]
Induction of VEGF Helps mobilize endothelial progenitors	Mice	GT3: [39]
Modulates gene expression pathways Oxidative stress, DNA damage, cell cycle, cell death, cell proliferation, hematopoiesis, blood vessel development	HUVEC cells	GT3: [28]

**Abbreviations**: gamma-tocotrienol (GT3); delta-tocotrienol (DT3); 3-hydroxy-3-methyl-glutaryl-coenzyme A reductase (HMG-CoA reductase); hematopoietic acute radiation syndrome (H-ARS); double-strand DNA break (dsDNA break); phosphorylated H2A histone family member X (γ-H2AX foci); primary human umbilical vein endothelial cells (HUVECs).

**Table 4:** Summary of observed radioprotective effects of tocotrienols in other target systems.

Other Organ Systems		
Major Radioprotective Effects for Tocotrienols	Model	References
Protected the lung in a thoracic-targeted radiation injury model (lung-PBI) Attenuated inflammatory lung tissue damage—alveolitis Beneficially modulated numerous cytokines, chemokines, and adhesion molecules Also protected the adjacent sternal bone marrow	Mice	GT3: [71]
Protected cardiac mitochondria against radiation damage in cell models and locally irradiated mouse heart Improved mitochondrial respiration, preserved mitochondrial membrane potential, and reduced MPTP opening However, organ-level cardiac function and structure were not protected	H9c2 cells Mice	DT3 and tocotrienol extracts [61,62]

**Abbreviations**: gamma-tocotrienol (GT3); delta-tocotrienol (DT3); partial body irradiation (PBI); mitochondrial permeability transition pore (MPTP); rat cardiomyocytes (H9c2 cells).

**Table 5:** Summary of the observed radioprotective signaling mechanisms for tocotrienols.

Signaling Mechanisms	Experimental Findings for Mechanistic Tocotrienol Radioprotection Research	Model	References
G-CSF induction	Both GT3 and DT3 strongly induce G-CSF within 24 h Required for hematopoietic recovery from neutropenia and H-ARS Required for 30-day survival against H-ARS G-CSF neutralizing antibody blocks efficacy	Mice	GT3: [37,45] DT3: [38]
Cytokines and chemokines induction	Both GT3 and DT3 induce circulating levels of interleukins (IL-1β, IL-6), other cytokines, and chemokines Modulates hematopoiesis Modulates inflammatory and immune responses in various systems Mobilizes progenitor and stem cells	Mice	GT3: [13,14,15,37,39,44,45] DT3: [38]
Anti-inflammatory action in tissues	**GI Tract:** DT3 reduced inflammatory markers in the GI tract (IL-1β and IL-6), NF-κB, and miR-30	Mice	DT3: [25,26]
	**Lungs:** GT3 reduced inflammatory markers and protected the lungs against inflammatory tissue damage	Mice	GT3: [71]
Progenitor mobilization	GT3 mobilizes hematopoietic, endothelial, and stromal progenitors Dependent on the induction of G-CSF, VEGF, and other cytokines Enables recovery of hematopoietic, mesenchymal, and endothelial systems	Mice	GT3: [39]
HMG-CoA reductase inhibition	Tocotrienols potently inhibit HMG-CoA reductase to provide vasculoprotective effects against radiation Protects against vascular oxidative and nitrosative stress Mevalonate, an HMG-CoA reductase product, blocks this effect HMG-CoA reductase inhibition induces the vasculoprotective factors thrombomodulin and eNOS	Mice	GT3: [12]
Thrombomodulin activation	GT3 activates endothelial thrombomodulin, which contributes to radioprotective efficacy 30-day survival and hematopoietic recovery after lethal TBI Mutant thrombomodulin reduces radioprotective efficacy Thrombomodulin activation is mediated through HMG-CoA reductase inhibition and eNOS	HUVEC cells Mice	GT3: [15,92]
Increasing tetrahydrobiopterin bioavailability	GT3 improves bioavailability of BH4, which is a cofactor for the vasculoprotective eNOS Radiation impairs BH4 bioavailability, which causes eNOS uncoupling and vascular oxidative stress This was prevented with GT3 treatment	Mice	GT3: [27]
DNA repair gene induction	GT3 induces DNA repair genes: *Rad50* induction protects against cytogenetic damage (dsDNA breaks) [29] Genomic analysis shows DNA repair gene clusters activated [28]	HUVEC cells	GT3: [28,29]
*Cebpd* induction	GT3 induces the transcription factor *Cebpd* Occurs in a G-CSF independent manner, which may involve NF-κB *Cebpd* is required for radioprotective efficacy for the hematopoietic and GI tract, and *Cebpd* is KO impaired: WBC recovery, particularly neutrophils Intestinal crypt colony survival Inflammatory and oxidative stress markers in GI tract Oxidative and nitrosative stress in GI tract	Mice	GT3: [21]
microRNAs	DT3 inhibits miR-30 levels in multiple cell types and tissues, which reduces apoptosis in hematopoietic cells Mediated through DT3 inhibiting the IL-1β/NF-κB pathways, which regulates miR-30 [26] GT3 has beneficial impacts on numerous microRNAs known to regulate the hematopoietic system’s response to radiation injury miR-125b, miR-15b, miR-34a, miR-92b, miR-150, miR-130a, and miR-143-3p [52] miR-30a, miR-126, and miR-375 [83]	Mice hCD34+ cells Primate	GT3: [52,83] DT3: [26]
Mitochondrial Function	Tocotrienols prevented radiation-induced mitochondrial respiratory dysfunction and uncoupling in cell models	H9c2 andHaCaT cells	DT3 and tocotrienol extract: [62]
	Tocotrienols prevented harmful mitochondrial changes in locally irradiated mouse heart Improved mitochondrial respiration, MPTP opening, mitochondrial membrane potential	Mice	tocotrienol extract: [61]

**Abbreviations**: gamma-tocotrienol (GT3); delta-tocotrienol (DT3); granulocyte colony-stimulating factor (G-CSF); hematopoietic acute radiation syndrome (H-ARS); gastrointestinal tract (GI-tract); interleukin (IL); nuclear factor kappa-light-chain-enhancer of activated B cells (NF-κB); microRNA (miR); vascular endothelial growth factor (VEGF); 3-hydroxy-3-methyl-glutaryl-coenzyme A reductase (HMG-CoA reductase); endothelial nitric oxide synthase (eNOS); total body irradiation (TBI); tetrahydrobiopterin (BH4); double-strand DNA break (dsDNA break); white blood cell (WBCs); mitochondrial permeability transition pore (MPTP); primary human umbilical vein endothelial cells (HUVECs); human CD34+ hematopoietic progenitor cells (hCD34+); rat cardiomyocytes (H9c2 cells); immortalized human epidermal keratinocytes (HaCaT cells).

## Data Availability

Not applicable.
